# Fact or fiction — Exploring resident mesenchymal stem cells in abdominal aortic aneurysm from multiple perspectives

**DOI:** 10.1016/j.gendis.2024.101210

**Published:** 2024-01-14

**Authors:** Shikai Shen, Yanshuo Han, Qingwei Gang, Mingyu Liu, Yao Qi, Jian Zhang

**Affiliations:** a Department of Vascular and Thyroid Surgery, The First Hospital of China Medical University, Shenyang, Liaoning 110001, China; b School of Life and Pharmaceutical Sciences, Dalian University of Technology, Panjin, Liaoning 124221, China

Mesenchymal stem cells (MSCs) are somatic adult stem cells derived from the mesoderm, possessing robust multipotency and regenerative properties. These cells are found in various tissues, including bone marrow, adipose tissue, the placenta, and the umbilical cord. The vascular wall serves as a reservoir for different types of stem and progenitor cells, contributing to tissue regeneration and vascular wall remodeling under pathological and environmental stress. Previous studies have reported the presence of MSCs or MSC-like cells in abdominal aortic aneurysm (AAA).^1^ However, due to the lack of exclusive and specific makers, identification and confirmation of vascular MSCs of patients with AAA remains challenging. Accurately determining the quantity and function of resident MSCs in patients with AAA holds immense importance in unraveling the pathogenesis and progression of the disease. Although Ciavarella and colleagues have successfully isolated arterial wall MSCs in patients with AAA,^2^^,^^3^ it is important to note that their findings have not been independently replicated in other centers. Therefore, the presence of vascular MSCs in patients with AAA requires validation using precise and powerful tools. Single-cell RNA sequencing (scRNA-seq) is an advanced technique that allows for the distinction and characterization of tissue stem cells. Despite the limited studies utilizing scRNA-seq on AAA samples, none have defined an MSC cluster. Thus, this investigation aimed to determine the plausibility of MSC existence in AAA samples using diverse methods.

We conducted scRNA-seq analysis on fresh AAA samples to investigate cellular heterogeneity and plasticity. A total of 36,750 qualified cells were obtained from six patients with AAA for further analysis. We identified 17 clusters and categorized them into 10 major cell types, including 6 immune cells and 4 non-immune cells, by merging similar gene expression clusters (Fig. 1A and, B). Unfortunately, we did not observe the presence of MSCs based on the classical makers proposed by the International Society for Cellular Therapy as the minimal criteria for defining multipotent MSCs.^4^ According to these criteria, MSCs must express high levels of CD90 (THY1), CD105 (ENG), and CD73 (NT5E) while lacking expression of CD34, CD45 (PTPRC), and CD14. Despite our efforts to identify MSC populations using this combination of markers, we were unsuccessful (Fig. 1C). After excluding the negative clusters, we observed a subset of mesodermal-derived cell groups that expressed some of the positive genes mentioned above. However, very few cell clusters simultaneously expressed CD90, CD105, and CD73 (Fig. 1D). Intriguingly, we found a significant enrichment of CD90^+^ cells in cluster 7, predominantly characterized by the expression of smooth muscle cell-related genes, such as “MYH11”, “MYL9”, “TAGLN”, and “ACTA2”. In contrast, CD73^+^ cells were mainly distributed in cluster 13, consisting of endothelial cells that co-expressed endothelial cell markers, including “CLDN5”, “ERG”, “VWF”, and “PECAM1”. Subsequently, we examined several genes associated with stem cell proliferation and multipotency (CSPG4, PDGFRB, CD34, PROM1, KIT, GLI1), considering the versatility of stem or progenitor cells. Although distinct clusters exhibited varying degrees of positive marker expression (Fig. 1E), we could not identify any specific group as MSCs based on single marker expression. For instance, KIT (CD117), a commonly used stem-cell maker, was eventually expressed by mast cells. Similarly, CD34^+^ cells were concentrated in the endothelial cell subgroup, while a subset of smooth muscle cells also exhibited PDGFRB^+^ cell expression (Fig. 1E). In order to maximize the identification of MSCs, we further performed the analysis with resolution = 1.0 (Fig. S4B, C) and subclustering of putative MSC groups (Fig. S4D, E). However, the results were still unsuccessful. Therefore, based on our scRNA-seq data, we do not have evidence to support the existence of MSCs in fresh adult AAA.

**Figure 1:**
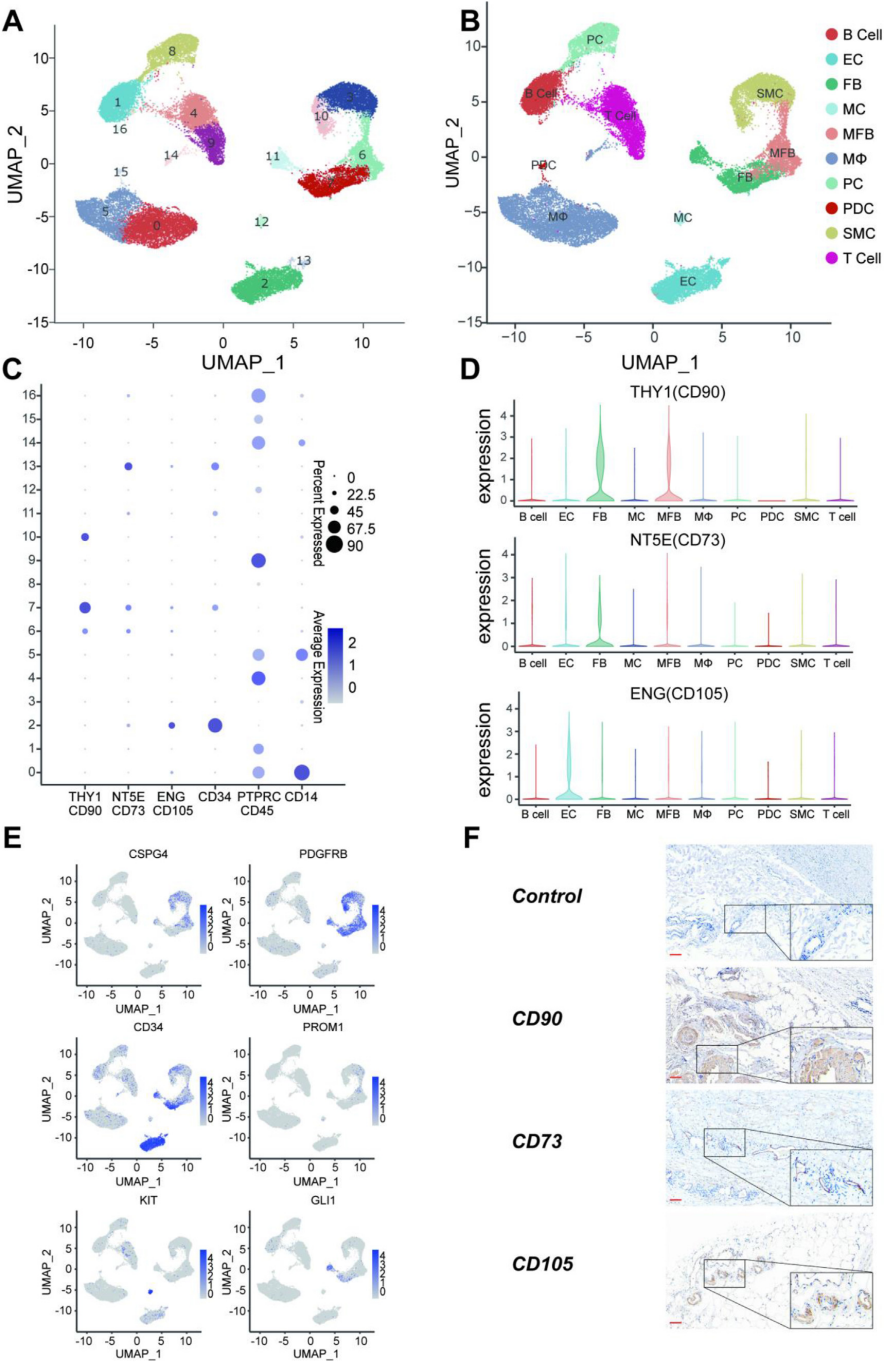
Clustering and characterization of freshly AAA and expression of MSC makers. **(A)** U-map analysis of obtained cells. **(B)** Identification and visualization of clusters. **(C)** The dotplot showing the expression of standard MSC makers in different subgroups. **(D)** The vinplot showing the expression of THY1 (CD90), NT5E (CD73), and ENG (CD105) in different clusters. **(E)** The distribution of common stem genes. **(F)** Representative immunohistochemistry staining image of AAA tissue. CD90^+^, CD73^+^, and CD105^+^ cells were concentrated in vascular endothelial (ECs) and smooth muscle cells (SMCs) of newly formed small blood vessels located in aneurysm adventitia. Scale bar = 100 μm. AAA, abdominal aortic aneurysm; MSC, mesenchymal stem cells.

Given the absence of MSCs in the scRNA-seq data, alternative strategies were pursued to examine the plausibility of MSC presence in AAA. While immunohistochemical staining of aneurysm tissue has been conducted in limited studies, the abundance (or lack) of MSCs in AAA samples has not been assessed using standard positive markers through serial section staining. Therefore, we performed immunohistochemistry on aneurysm serial specimens of 12 cases (among whom 6 were also subjected to scRNA-seq) to characterize the MSC population using primary antibodies CD90, CD73, and CD105. Consistent with scRNA-seq data, little or no evidence demonstrated the presence of MSCs expressing all three positive makers synchronously. Meanwhile, cells exhibiting hyperexpression of CD90, CD73, or CD105 were primarily located in the adventitial vasa vasorum, particularly within endothelial or smooth muscle cells of neonatal capillaries (Fig. 1F). This observation was consistent with scRNA-seq analysis, which identified certain mesodermal-derived cell subgroups expressing typical MSC makers. To validate the reliability of this approach, we also performed the same staining on tissues known to be abundant in MSCs, such as the umbilical cord, with representative results presented in Figure S2. Meanwhile, to further validate our results, we employed multiplexed immunohistochemistry staining of CD90, CD73, and CD105 merged with DAPI (4′,6-diamidino-2-phenylindole), but did not find any triple-positive cells. Consistent with the results of conventional immunohistochemistry, we also identified cells with high expression of CD90, CD73, or CD105 in the adventitia of blood vessels (Fig. S5).

Furthermore, attempts were made to isolate and culture AAA-MSCs *in vitro* using an established enzymatic method.^2^^,^^3^ However, the results were inconsistent and non-reproducible. Intriguingly, MSC-like cells were obtained at the junction of the adventitia and perivascular adipose tissue (Fig. S3B). Flow cytometric analysis confirmed positive expression of CD73, CD90, and CD105, while CD34, CD45, and HLA-DR were negative (Fig. S3C). *In vitro* induction experiments revealed that the cells possessed the ability to differentiate into osteoblasts, chondrocytes, or adipocytes (Fig. S3D). Therefore, inadequate clearance of perivascular adipose and fibrous tissue may lead to contamination of stem cells from the perivascular tissue. These findings indicate that the methods and techniques employed in our study were not limiting factors, and the acquisition of MSCs appears to be tissue-specific.

In summary, our research explored the possibility and plausibility of MSCs in AAA tissue. Unexpectedly, our results did not provide direct evidence supporting the existence of MSCs. Notably, the scRNA-seq data analysis revealed the presence of six primary non-immune cell types in adult human AAA, while no MSCs were identified. However, the expression of stem cell markers was observed in specific mesodermal populations, including smooth muscle cells, myofibroblasts, and endothelial cells, as evidenced by both scRNA-seq data and immunohistochemistry results from consecutive sections. Although previous research indicated the presence of stem cells in AAA patients, the markers chosen by Ryer et al were controversial,^1^ and are not universally recognized as classic markers for MSCs. Based on our findings, we speculate that the lack of MSCs in our samples may be attributed to the end-stage nature of the arterial aneurysm specimens. At this stage, resident vascular stem cells may have completed their differentiation and entered an irreversible state, thereby hindering their potential contribution to further repair tissue. In addition, even if specific subpopulations were isolated to obtain MSCs, their low quantity might still limit their potential to exert a significant effect during this phase, considering our subgroup analysis did not identify any MSCs or MSC-like cells. On the other hand, although previous scRNA-seq analysis of thoracic aortic aneurysm patients has identified subpopulations of MSCs,^5^ it is important to consider the different origins of thoracic and abdominal aortas in terms of genetics, as well as the varying types of perivascular adipose tissues. These variations may contribute to differences in the distribution and residency of stem cells. This partially explains why infrarenal AAA has a higher occurrence rate in clinical settings, as there are scarce stem cells available to facilitate repair and anti-inflammatory functions. These findings provide valuable insights into the recognition and localization of mesenchymal stem cells in large vessel diseases and arouse further discussion on the underlying mechanisms of their actions.

## Ethics declaration

The studies involving human participants were reviewed and approved by the Ethics Committee of the First Affiliated Hospital of China Medical University (The functional disturbance and restoration of mesenchymal stem cell in abdominal aortic aneurysm is regulated by crosstalk between SIRT1/FOXO1 and TLR-4/PI3K/Akt pathway. No. AF-SOP-07-1.1-01, 2019/03/06). All participants have agreed to participate in the experiment and have signed informed consent forms.

## Author contributions

S.S., J.Z., and Q.G. designed the research. M.L., Y.H., and S.S. performed the research and analyzed the data. S.S. and Y.Q. wrote the paper. The authors read and approved the final manuscript.

## Conflict of interests

The authors declare no competing interests.

## Funding

This research was supported by the National Natural Science Foundation of China (No. 82170507, 81970402, 81800407).

## Data availability

All the results are presented in the article and supplementary material. Further inquiries can be directed to the corresponding author.
