# Supplementary material for: Fact or fiction — Exploring resident mesenchymal stem cells in abdominal aortic aneurysm from multiple perspectives
Source: Genes Dis. 2024 Jan 14;12(1):101210. doi: 10.1016/j.gendis.2024.101210 (PMC11472224; doi:10.1016/j.gendis.2024.101210)
Supplement: Multimedia component 1 [file mmc1.docx]

**Additional File 1: Methods used in the study**

**Methods:**

**Sample collection and preparation**

The freshly collected tissue samples were washed with sterile phosphate-buffered saline (PBS) and stored in the preservation solution(Seekone, China) for freezing within 30 minutes after surgery. The perivascular connective tissue and adipose tissue were meticulously excised. For each sample, a piece of aortic tissue (1-2 cm^2^) was separated into thin layers and cut into small pieces in DMEM (Gibco, USA) with 10% fetal bovine serum (FBS). Then, the small pieces of tissue were placed into Seekone digestion solution (Seekone, China) for 30-45 minutes, which mainly contained collagenase type II (Sigma, USA), collagenase type IV (Sigma, USA), Seekone protease E (Seekone, China), Seekone protease G (Seekone, China), Seekone protease H (Seekone, China), hyaluronidase type I (Sigma, USA), and Hanks’ balanced salt solution (HBSS, Sigma, USA). After 2-3 rounds of digestion in a 37°C water bath, the tissue was completely digested. Then, we filtered the cells with a 70-μm cell filter and centrifuged them at a speed of 300-400 g at 4°C for 5-6 minutes. After removing erythrocytes (Miltenyi, USA), the decision to perform debris and dead cell removal was made (Miltenyi, USA). Cell count and viability were estimated using the fluorescence Cell Analyzer (Countstar® Rigel S2) with AO/PI reagent serum albumin. Finally, the fresh cells were washed twice in DMEM and then resuspended at a concentration of 1×10^5^ cells per ml in HBSS and 2% FBS.

**Single RNA sequence and data analysis**

To construct the scRNA-seq libraries, single-cell suspensions, at a concentration of 1 × 10^5^ cells/mL, were loaded onto microfluidic devices, and the GEXSCOPE Single-Cell RNA Library Kit (Singleron, 1110011) was used following the protocol provided by Singleron GEXSCOPE^[1]^. After quality checks, individual libraries were diluted to a concentration of 4 nM and combined for sequencing on the Illumina NovaSeq 6000 platform using 150-bp paired-end reads. The resulting gene-cell expression matrix was utilized for further analysis in Seurat within R version 4.3.1. Cells were screened based on the following criteria: gene counts ranging between 200 to 7,000, UMI counts below 30,000, and exclusion of cells with more than 10% mitochondrial content.

Subsequently, Seurat’s Clustree function was used to evaluate the relatively suitable resolution, the FindClusters function was implemented with a clustering analysis resolution parameter of 0.5. The resulting clusters were visualized using either t-Distributed Stochastic Neighbor Embedding (t-SNE) or Uniform Manifold Approximation and Projection (UMAP). Each cluster was annotated with a cell type using known marker genes. Finally, Seurat’s FeaturePlot and VlnPlot functions were used to visualize the expression of specific genes in our analysis.

**Immunohistochemical staining**

For immunohistochemical analysis, paraffin-embedded tissue sections were deparaffinized, rehydrated, and underwent antigen retrieval in sodium citrate buffer (pH=6.0) using a pressure cooker. Tissue sections were then blocked with 5% bovine serum albumin (BSA) and incubated overnight with primary antibodies (CD90, Proteintech, DF4804; CD73, Abcam, ab133582; CD105, Proteintech, 10862-1-AP) at 4°C. After washing with PBS, the tissue sections were incubated with HRP-conjugated secondary antibodies(1:2000, Abcam, ab205718) and developed with diaminobenzidine(DAB) ubstrate. Tissue sections were counterstained with hematoxylin and mounted with a coverslip. Negative controls were prepared using PBS. Samples were examined and imaged under a microscope(C2+ system, Nikon). The procedure was repeated six times.

**Multiplexed immunohistochemistry**

To perform multiplexed immnohistochemistry(mIHC) staining, an AlphaTSA Multiplex IHC kit (Alpha X, China) was utilized. Initially, the concentration and order of CD90(Proteintech, DF4804), CD73(Abcam, ab133582) and CD105(Proteintech, 10862-1-AP) were optimized. The slides were subjected to deparaffinization and dehydration using xylene and ethanol (various concentrations), followed by antigen retrieval via microwave. Following a 15 minute incubation with freshly prepared 3% H_2_O_2_. Subsequently, the tissues were incubated with primary antibody overnight at 4°C. Then the spicemens were incubated with secondary-HRP (abcam, USA), and AlphaTSA working solution (Alpha X, China). Finally, the slides were incubated with DAPI (abcam, ab104139). The Pannoramic DESK Scanner (3D HISTECH, Hungary) was employed to scan all the slides, and the images were analyzed using the CaseViewer software (3D HISTECH, Hungary).The procedure was repeated six times.

**Cell isolation and culture**

Samples of AAA, with or without perivascular tissue, were rinsed thoroughly with PBS to remove blood from the sample. Subsequently, the samples were cut into small fragments approximately 2 mm long and digested for 2 hours with type II collagenase (Gibco) at a concentration of 1 mg/ml. The digested tissue was filtered through 70-μm strainers (BD Biosciences) and centrifuged at 300g for 5 minutes. The cell pellets were seeded into DMEM supplemented with 10% Fetal Bovine Serum (FBS; SIGMA) for further incubation. Our study selected cells between the 3rd and 5th passages (P3-P5) for further identification.

**Flow cytometry**

Flow cytometry was utilized for MSC identification. The cells were collected at a density of 1×10^^6^ cells per tube. Primary antibodies were added and incubated in darkness at 4°C for 30 minutes. The extensive panel of conjugated antibodies used in this experiment included anti-human CD90-PE (328109, BioLegend), anti-human CD73-FITC (344015, BioLegend), anti-human CD105-APC (800507, BioLegend), anti-human CD34-PE (343505, BioLegend), anti-human CD45-FITC (304005, BioLegend), and anti-human HLA-DR (307609, BioLegend). Negative controls were performed using appropriate irrelevant conjugated antibodies. Analysis was carried out using a flow cytometer (DxFLEX, Beckman-Coulter, USA). The results were analyzed using Flowjo X software.The procedure was repeated three times.

**Multilineage differentiation of obtained MSCs**

To induce adipogenic differentiation, MSCs were seeded at a density of 5× 10^^4^ cells/well into a six-well plate and incubated with 2 ml/well adipogenesis induction medium (Cyagen Biosciences Inc., GUXMX-90031) in a warm and humid envrionment (37^0^C incubator, 5% CO_2_) for 15days. The cells were fixed with 4% paraformaldehyde for 30 minutes, and then were stained with 2 ml/well of Oli Red working solution (Cyagen, OILR-10001) . After incubating for 30 minutes, the samples were observed under a microscope.The procedure was repeated three times.

To induce osteogenic differentiation, MSCs were seeded at a density of 5× 10^4 cells/well into a six-well plate and incubated with 2 ml/well osteogenic induction medium (Cyagen Biosciences Inc., GUXMX-90041) in a 5% CO_2_-/water-saturated incubator at 37^o^C for 15 days. The cells were fixed with 4% paraformaldehyde for 30 minutes, and and then were stained with 2 ml/well of Alizarin Red S working solution (Cyagen, ALIR-10001) .After incubating for 10 minutes, the samples were observed under a microscope.The procedure was repeated three times.

To induce chondrogenic differentiation, 5×10^5 MSCs were pelleted in a 15 mL polypropylene tube and incubated in 500ul Chondrogenic Differentiation Medium (Cyagen Biosciences Inc., GUXMX-90021) for 48h. After being seeded, the cells were given fresh complete chondrogenic induction medium every 2-3 days until 21 days, when approximately 2mm of cartilaginous spheres had formed. Finally, cartilaginous spheres were fixed with 4% paraformaldehyde for 30 minutes,embedded in paraffin using standard procedures, and stained with Alcian Blue 8GX Solultion (Cyagen, ALCB-10001

) for visualization.The procedure was repeated three times.

**Reference**

1. Dura B, Choi JY, Zhang K, et al. scFTD-seq: freeze-thaw lysis based, portable approach toward highly distributed single-cell 3' mRNA profiling. Nucleic Acids Res. 2019;47(3):e16. doi: 10.1093/nar/gky1173.
