# Supplementary material for: Fact or fiction — Exploring resident mesenchymal stem cells in abdominal aortic aneurysm from multiple perspectives
Source: Genes Dis. 2024 Jan 14;12(1):101210. doi: 10.1016/j.gendis.2024.101210 (PMC11472224; doi:10.1016/j.gendis.2024.101210)
Supplement: Multimedia component 2 [file mmc2.docx]

| **Table S1: Cell-cluster-specific marker genes of different cell types in AAA** | | |
| --- | --- | --- |
| **Cell types** | **Abbreviations** | **Marker genes** |
| Macrophages | Mø | CD86, CD68, LYZ, CD14, FCGR3A, CD1C |
| B cells | B cells | *MS4A1, CD79A, CD79B* |
| Endothelial cells | EC | CDH5, CLDN5, ERG, VWF, PECAM1 |
| Smooth muscle cells | SMC | MYH11, MYL9, TAGLN, ACTA2 |
| T cells | T cells | CD3D, CD3E, CD3G, TRBC1, TRBC2, TRAC |
| Myofibroblasts | MFB | TAGLN, ACTA2,COL1A1, COL1A2, LUM |
| Fibroblasts | FB | DCN, COL1A1, COL1A2, COL3A1, LUM |
| Plastma cells | PC | MZB1, SDC1, LY6D, JCHAIN |
| Mast cells | MC | CPA3, KIT, MS4A2 |
| Plasmacytoid dentritic cells | PDC | CD4, LILRA4, GZMB |

**Supplementary Table S1 Cell-cluster specific marker genes of different cell types**
