# Supplementary material for: Fact or fiction — Exploring resident mesenchymal stem cells in abdominal aortic aneurysm from multiple perspectives
Source: Genes Dis. 2024 Jan 14;12(1):101210. doi: 10.1016/j.gendis.2024.101210 (PMC11472224; doi:10.1016/j.gendis.2024.101210)
Supplement: Multimedia component 4 [file mmc4.docx]

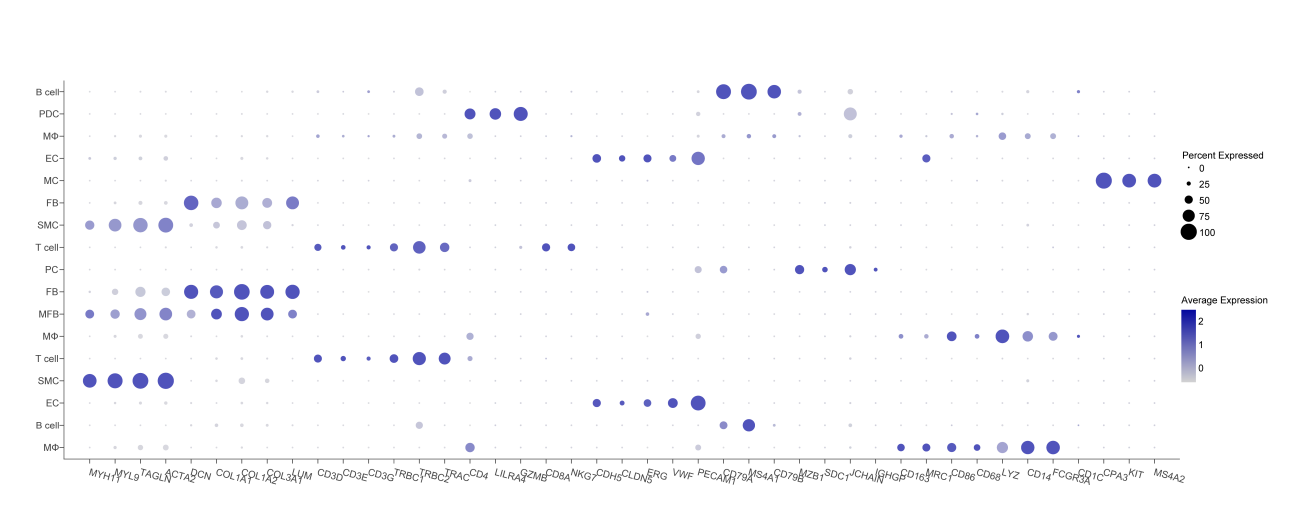


**Supplementary Figure S1** Dotplot showing specific marker genes expression of different cell types

**Abbrevialations**

EC: endothelial cell; FB: fibroblast; MC: mast cell; Mø: macrophage; MFB: myofibroblast; PC: plasma cell; PDC: plasmacytoid dendritic cell; SMC: smooth muscle cell
