# Supplementary material for: Fact or fiction — Exploring resident mesenchymal stem cells in abdominal aortic aneurysm from multiple perspectives
Source: Genes Dis. 2024 Jan 14;12(1):101210. doi: 10.1016/j.gendis.2024.101210 (PMC11472224; doi:10.1016/j.gendis.2024.101210)
Supplement: Multimedia component 5 [file mmc5.docx]

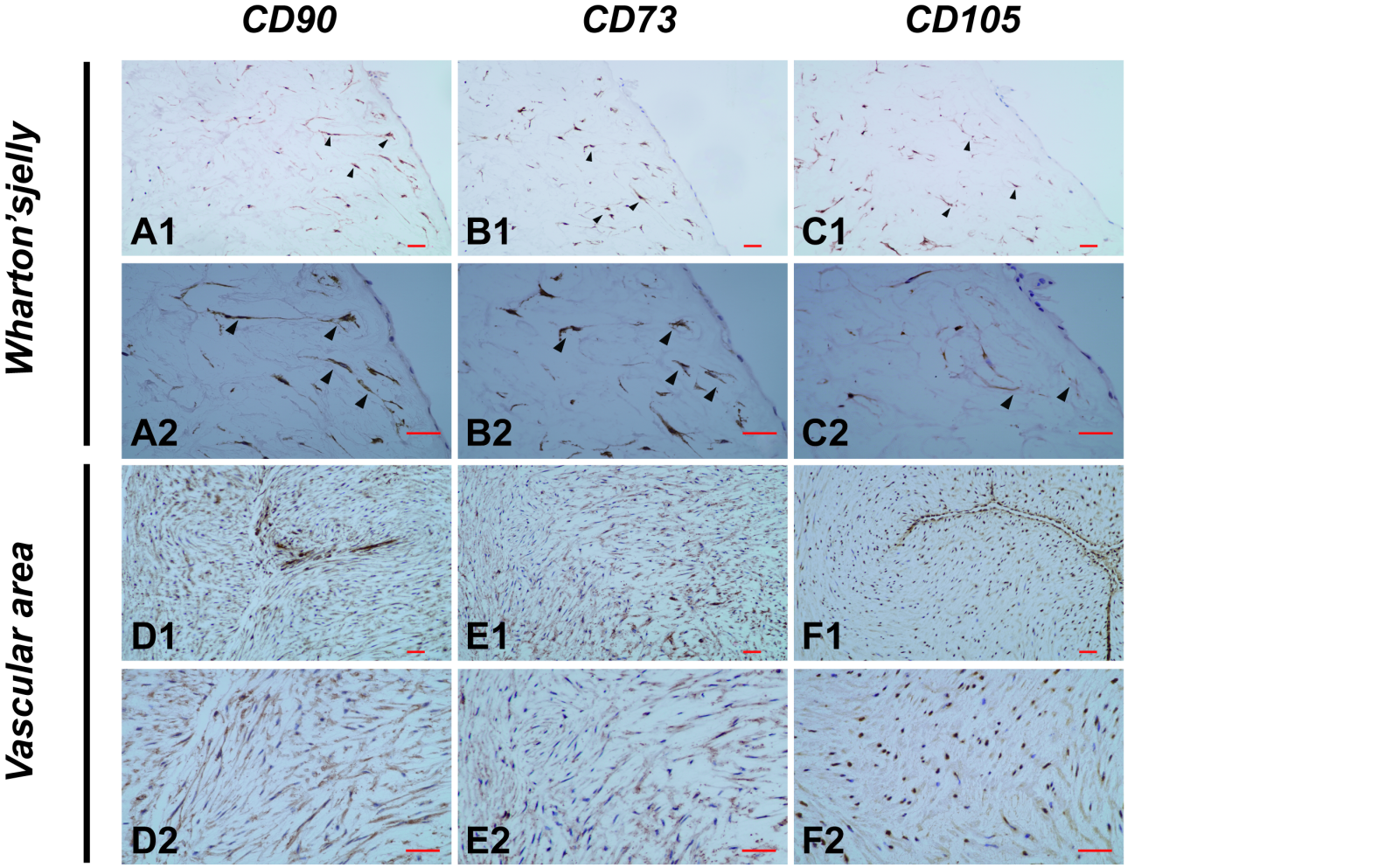


**Supplementary Figure S2** Representative immunohistochemical staining of 4μm thick sections obtained from umbilical cord tissues. **A1-C2** CD90, CD73 and CD105 positive cells in Wharton’s jerry of serial sections. Arrows showing co-expression of CD90, CD73 and CD105 cells. Original magnifification, ×200 in A1–C1, × 400 in A2–C2. **D1-F2** Representive high expression CD90, CD73 and CD105 cells in vascular area. Original magnifification, ×200 in D1–F1, × 400 in D2–F2. Scale bar represents 10μm.
