# Supplementary material for: Fact or fiction — Exploring resident mesenchymal stem cells in abdominal aortic aneurysm from multiple perspectives
Source: Genes Dis. 2024 Jan 14;12(1):101210. doi: 10.1016/j.gendis.2024.101210 (PMC11472224; doi:10.1016/j.gendis.2024.101210)
Supplement: Multimedia component 6 [file mmc6.docx]

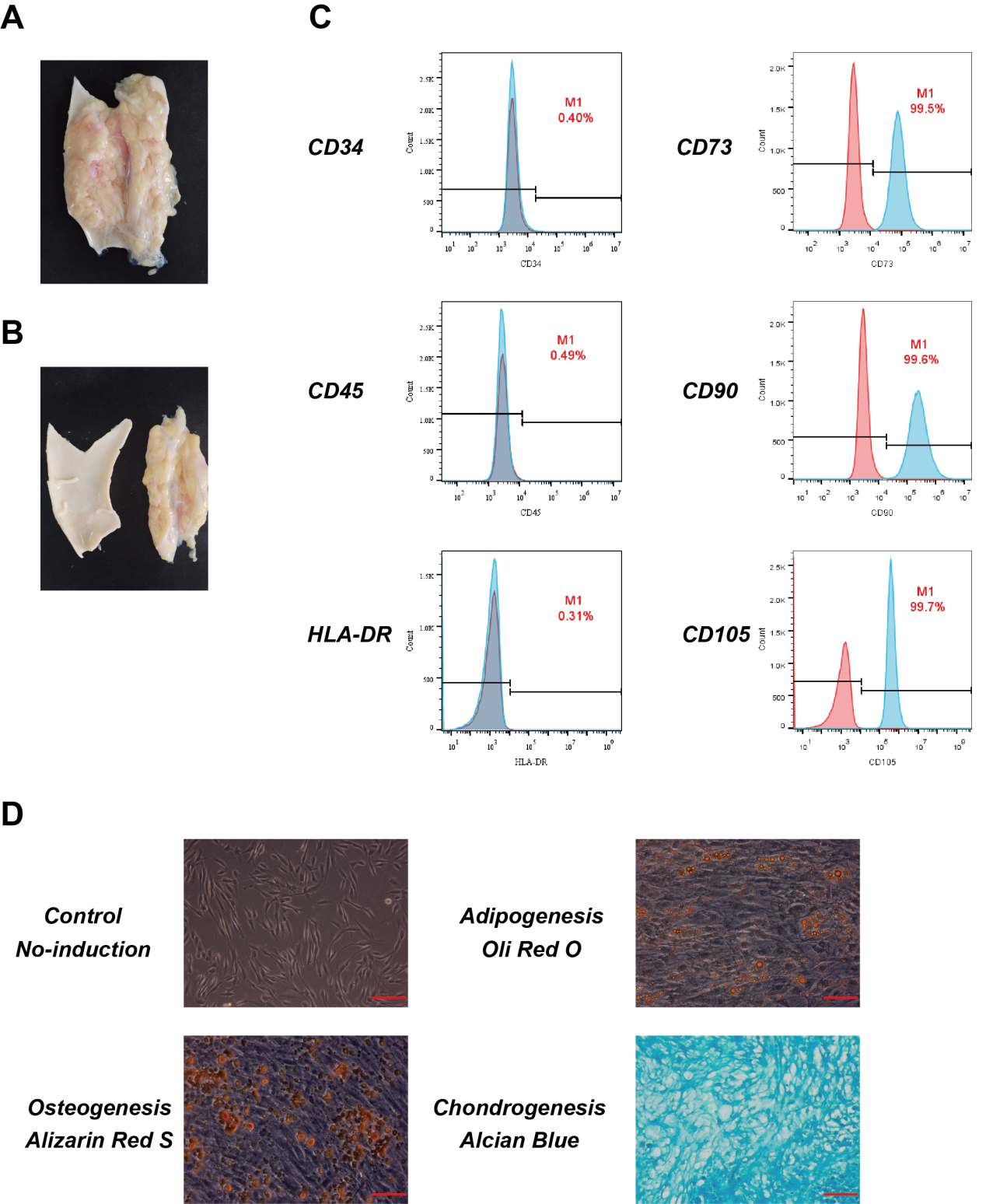


**Supplementary Figure S3** Identifiaction of cultured human MSCs at the junction of the adventitia and perivascular adipose tissue in AAA. **A** Fresh abdominal aortic samples obtained from clinical cases. **B** Representive aortic wall (left) and perivascular tissue (right). **C** Flow cytometry analysis of cell markers on the cultured MSCs. **D** Representative images of the 3^rd^ generation MSCs and their trilineage differentiation into osteoblasts, adipocytes, and chondrocytes. Scale bar = 20μm.
