# Supplementary material for: Fact or fiction — Exploring resident mesenchymal stem cells in abdominal aortic aneurysm from multiple perspectives
Source: Genes Dis. 2024 Jan 14;12(1):101210. doi: 10.1016/j.gendis.2024.101210 (PMC11472224; doi:10.1016/j.gendis.2024.101210)
Supplement: Multimedia component 7 [file mmc7.docx]

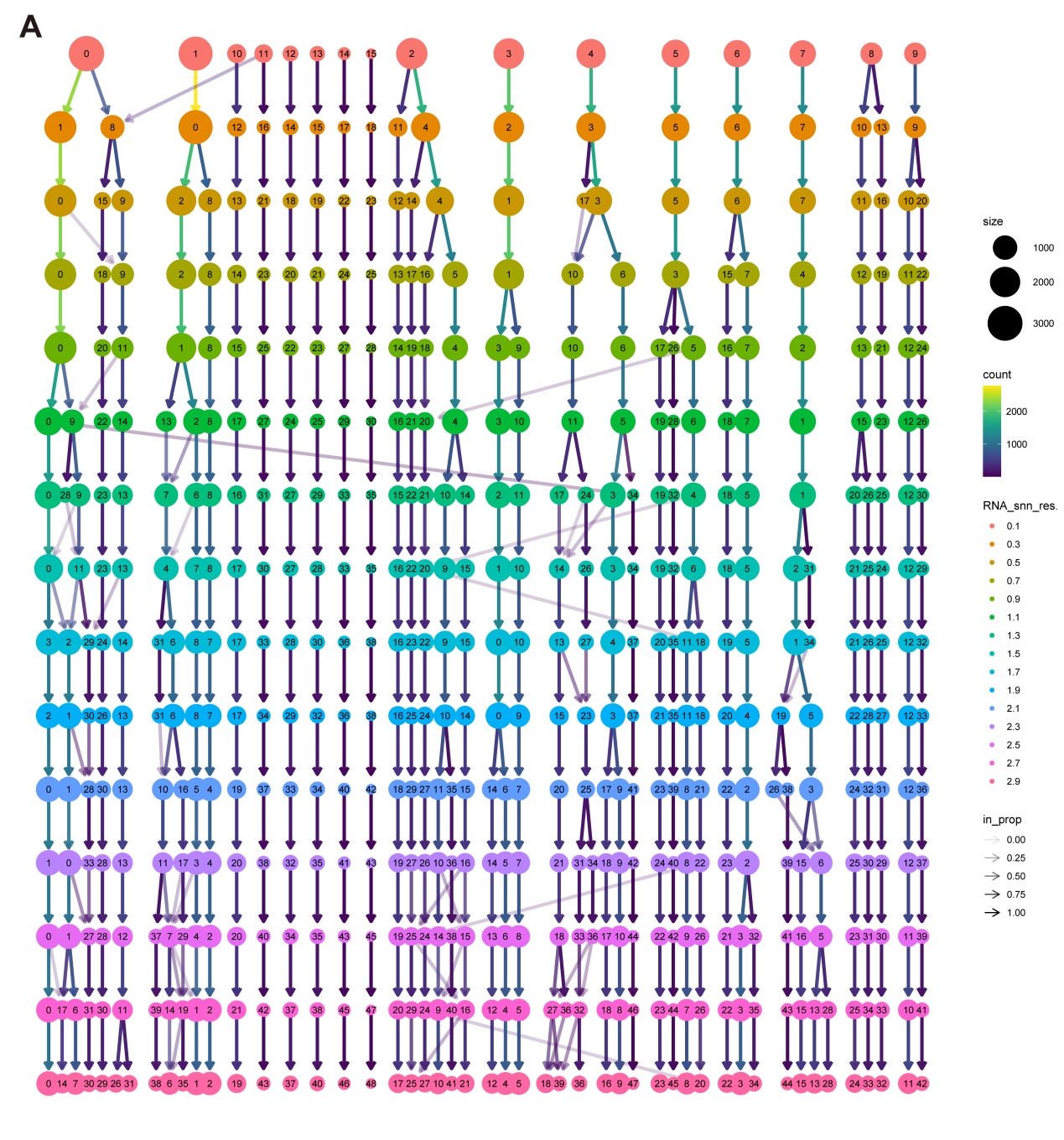


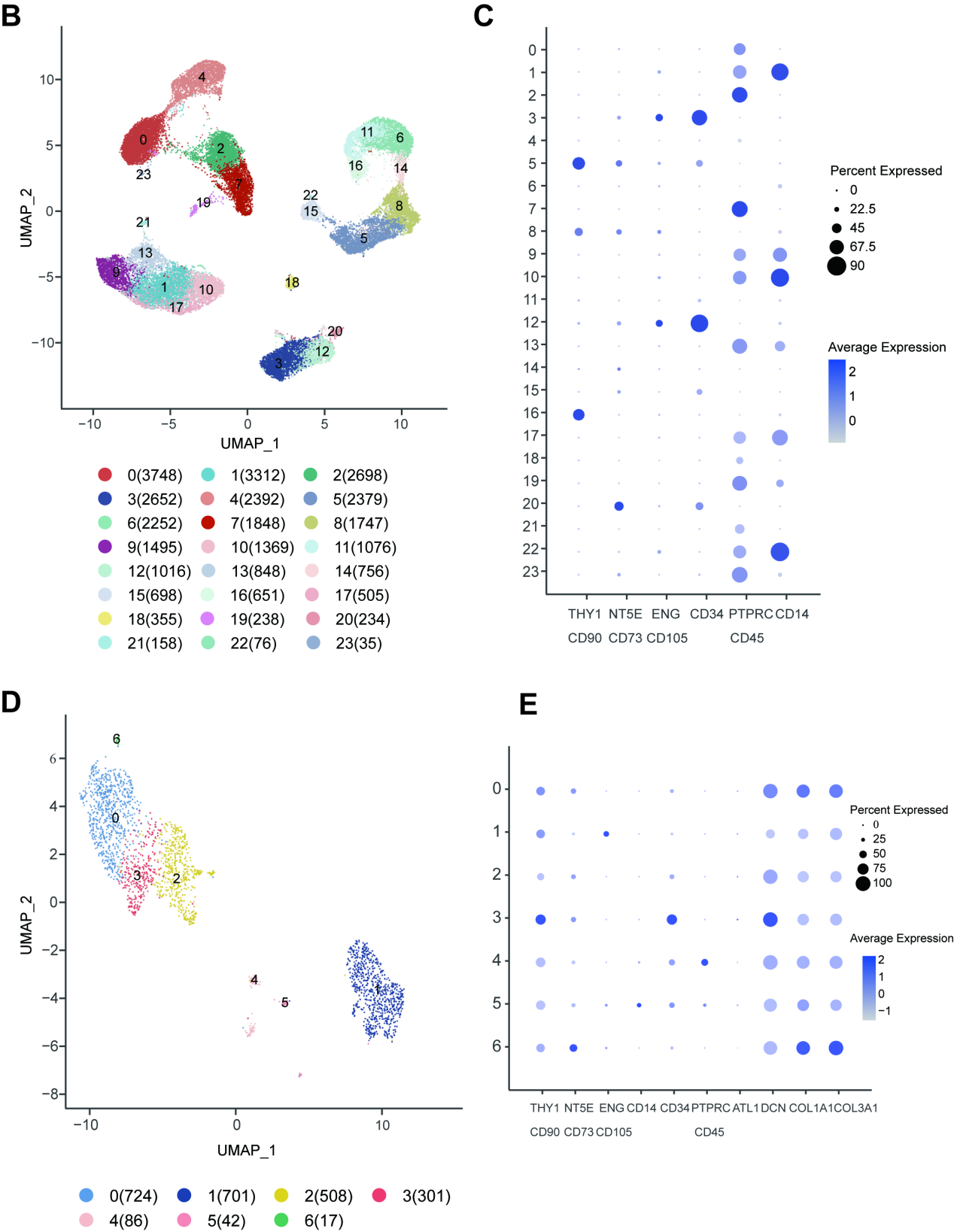


**Supplementary Figure S4** Different resolutions and subclustering analysis of sc-RNA seq data. **A** Clustree function to evaluate the relatively suitable resolution. **B** U-map analysis to obtain 23 different clusters (resolution=1.0). **C** Dotplot showing the expression of standard MSC makers in different clusters (resolution=1.0) **D** Sub-clustering of the 5^th^ group that were suspected to be MSCs. **E** Dotplot showing the expression of MSCs(THY1, NT5E, ENG, PECAM1, CD34, PTPRC) and fibroblasts(DCN, COL1A1, COL3A1) in subgroup analysis.
