# Supplementary material for: Fact or fiction — Exploring resident mesenchymal stem cells in abdominal aortic aneurysm from multiple perspectives
Source: Genes Dis. 2024 Jan 14;12(1):101210. doi: 10.1016/j.gendis.2024.101210 (PMC11472224; doi:10.1016/j.gendis.2024.101210)
Supplement: Multimedia component 8 [file mmc8.docx]

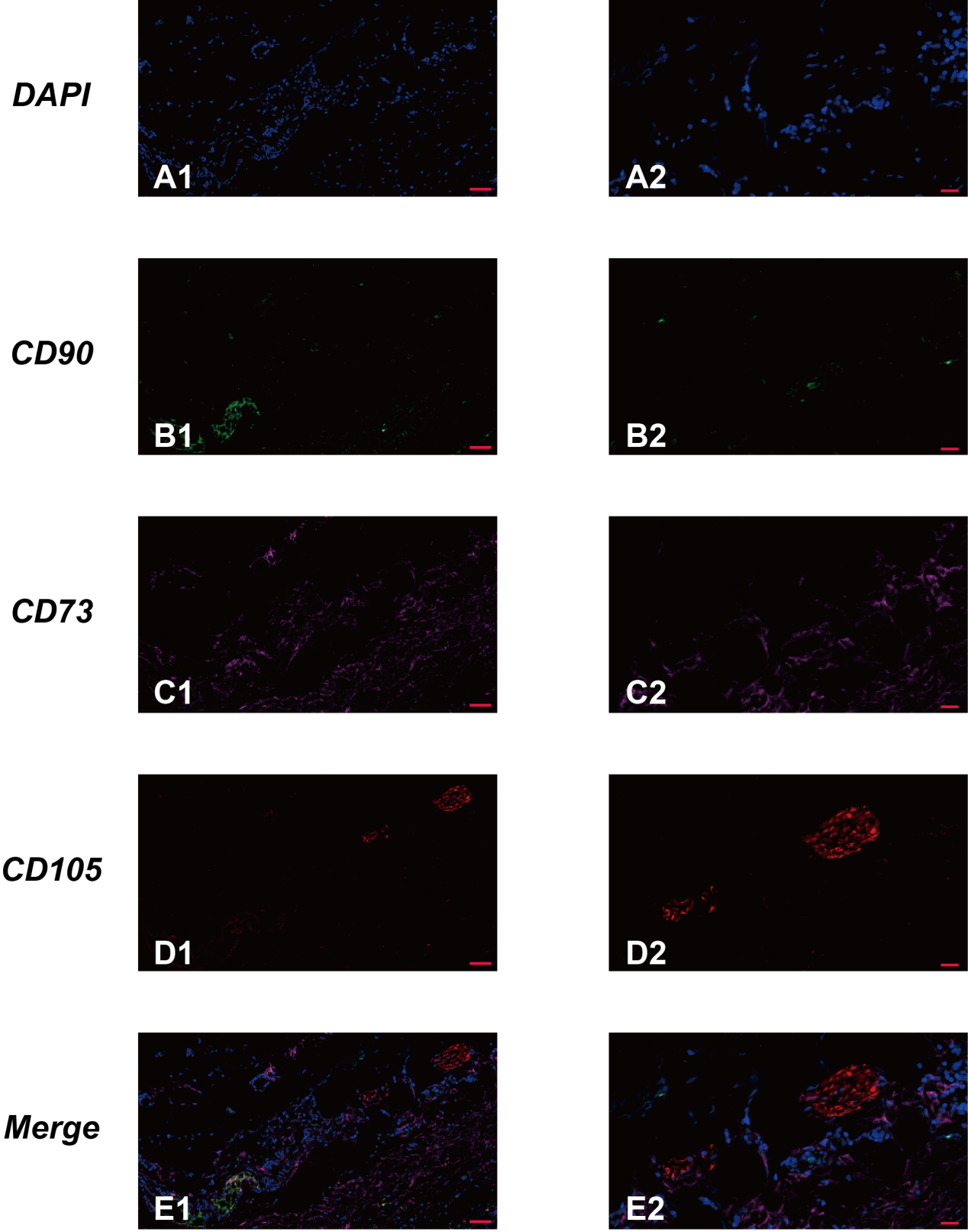


**Supplementary Figure S5** Representative immunofluorescent staining of MSC positive markers merged with DAPI in AAA samples. **A1-E1** DAPI, CD90, CD73 and CD105 positive cells in AAA tissue. Original magnifification, ×200. **B1, D1** CD90^+^and CD105^+^ cells are concerntrated in SMCs of small blood vessels locating in aneurysm adventitia, bar=50μm. **A2-E2** DAPI, CD90, CD73 and CD105 positive cells in AAA tissue. **B2, D2** CD90^+^and CD105^+^ cells are concerntrated in SMCs of small blood vessels locating in aneurysm adventitia, bar=25μm.
